# The effect of pole length on physiological and perceptual responses during G3 roller ski skating on uphill terrain

**DOI:** 10.1371/journal.pone.0211550

**Published:** 2019-02-22

**Authors:** Per-Øyvind Torvik, Erna Dianne von Heimburg, Torkel Sende, Boye Welde

**Affiliations:** 1 Department of Sports Sciences and Physical Education, Nord University, Levanger, Norway; 2 UiT The Arctic University of Norway, School of Sport Sciences, Tromsø, Norway; Universita degli Studi di Verona, ITALY

## Abstract

The benefits of using longer than self-selected poles have been shown in double poling, but these potential benefits have not been examined in the gear 3 ski skating sub-technique (G3), during which the poling movement is very similar to double poling. The aim of this study was to examine the effect of longer than self-selected poles on physiological and perceptual responses in the G3 sub-technique. Ten cross-country skiers and biathletes (VO_2max_ 72.4 ± 3.0 ml∙min^-1^∙kg^-1^, age 20.1 ± 2.8 years, height 1.81 ± 0.03 m and weight 73.1 ± 4.6 kg) completed two tests, each with three different submaximal intensities, during roller skiing using the G3 technique. The first test was carried out at a fixed speed (10 km∙h^-1^) and the skiers performed two intervals of 5 min at 7, 9 and 11% inclination on a roller ski treadmill with self-selected poles (SSP) and 7.5 cm longer poles (LP) at each step. The second test had a fixed inclination of 4% and speeds of 14, 17 and 20 km∙h^-1^, also performed with SSP and LP at each step. At fixed speed, the oxygen uptake was 2.7% lower (P = 0.005) and the gross efficiency (GE) 2.1% higher (P = 0.01) with LP than with SSP at the steepest inclination of 11%. At fixed inclination, the oxygen uptake was 2.1% lower (P = 0.01) and the GE was 4.1% higher (P = 0.03) with LP than with SSP at the highest speed of 20 km∙h^-1^. At 14 km∙h^-1^, the oxygen uptake was 3.0% lower (P = 0.05) and GE was 3.8% higher (P = 0.03) with LP than with SSP. Our novel findings show that longer poles in the G3 technique may enhance the efficiency of skiing.

## Introduction

Effectively utilising metabolic energy to produce high speed is a crucial factor for endurance performance in sports like cross-country (XC) skiing [1]. The constant change in workload in XC skiing due to varying track conditions (changing snow and weather conditions) and track profiles consisting of different types of terrain (flat, uphill, downhill) challenge athletes with respect to the use of different sub-techniques and types of muscle use that require major adaptability of the cardiovascular system [1]. The speed and technique on the uphills is of particular interest since ~50% of race time is spent there [2, 3], and the main time differences between skiers have been reported to occur during uphill skiing [4, 5].

In the 1980s, XC skiing went through a technique revolution with the development of the skating style and five different ski skating sub-techniques are currently identified and used as a functional gear system during training and competitions [2, 6, 7]. Already in the very beginning of the world cup ski skating races, in 1985, longer poles than in classic style (~7.5–10 cm) were found beneficial [8]. The gear 3(G3) ski skating sub-technique is traditionally used at a high speed and is a symmetric sub-technique with one parallel pole plant with each skating stroke, one on each side [6]. The similarity in upper body work between double poling (DP) and G3 [9, 10] forms the basis for the claim that these two sub-techniques are limited by almost the same factors, at least when considering the work of the upper body muscles. The similarities between G3 and DP are shown in the way potential energy is gained between pole plants, the propulsive force in the poling action, and the conformity in upper-body muscle work [9].

Previous research has shown the beneficial effects of using longer poles (self-selected + 5–10 cm) in DP [7, 11, 12, 13, 14]. During DP, the force is transferred to the ground via the poles, and pole length seems to have a crucial influence on VO_2_-cost and performance during DP in classic skiing [11]. The missing information about the effect of longer poles in skating necessitate a comparison with results of DP. Longer poles in DP enable higher speeds both in flat and level terrain [11] and there might be an inverted U-shape relationship between pole length and performance [13]. Several DP studies [11, 12] have pointed out the reduced vertical displacement of the body centre of mass (COM) while using longer poles as one important factor for lowered VO_2_-cost and improved performance. The reduced VO_2_-cost and improved performance with longer poles is also explained by the longer poling time and the effectiveness of slower muscle contraction [11]. Longer poles in DP lead to a more upright working position, reduced distance between COM and the poles, a pole plant further behind which provides a better working posture and a reduced VO_2_-cost at the same workload [12]. Further, longer poles produce greater propulsive force, allow the skier to use the upper body and body mass more effectively [12,15] and, as pointed out by Carlsen and colleagues [12], longer poles and an upright posture will reduce the total range of motion on steeper terrain.

Despite the beneficial effects observed in the use of longer poles in DP, and the fact that skiers in skating are allowed to use poles as long as their body height (FIS§343.8.2), the potential benefit of longer poles in skating has not been fully explored. Therefore, the main aim of this study was to investigate the effect of pole length on physiological and perceptual responses caused by increasing speed and inclination during submaximal G3 roller skiing. It was hypothesised that longer poles have lower VO_2_-cost and higher skating efficiency during submaximal G3 uphill treadmill roller skiing when workloads were altered either by inclination or by speed.

## Materials and methods

### Participants

Ten highly-trained, male junior skiers (six XC skiers and four biathlon skiers) with uphill treadmill running maximal oxygen uptake and heart rate (HR) of (mean ± SD) 72.4 ± 3.0 ml ∙ min^-1^∙ kg^-1^ and 196 ± 5 beats∙min^-1^, and age, height and body mass of 20.1 ± 2.8 years, 1.81 ± 0.03 m, and 73.1 ± 4.6 kg, respectively, volunteered to participate in this study. The participants were students at a Norwegian high school or a university with a special programme for XC skiing and biathlon. The participants had 147 ± 83 FIS points at the start of the study, and they had competed at the national level for 4 ± 2 years (range 2–8 years). They were familiar with treadmill roller skiing. The participants provided written informed consent to participate in the study, which was pre-approved by the Norwegian Centre for Research Data and the Regional Ethics Committee in Trondheim, Norway, according to the Declaration of Helsinki. All participants were 18 years or older at the start of the study.

### Design

To study the effect of self-selected pole length (SSP) and longer pole length (LP) (SSP+7.5 cm) on physiological and perceptual responses in the G3 technique during uphill treadmill roller skiing, two submaximal incremental tests with fixed speed or fixed inclination using a cross-over design were implemented. To determine submaximal intensity at each step of the two submaximal protocols, a peak oxygen uptake (VO_2peak_) test in the G3 technique was carried out 2–7 days before the submaximal tests. A pilot study was conducted to determine inclination and speed for the two submaximal protocols, according to the intensity zones for endurance training established by the Norwegian Olympic Sports Centre (Olympiatoppen) [16]. The three lowest intensity zones for endurance training were established individually for each skier to be used as submaximal intensities in this study, namely < 65% (zone 1), 65–79% (zone 2) and 80–87% (zone 3) of the subjects’ skating VO_2peak_. Participants that measured values higher than 87% of their skating VO_2peak_ during the submaximal protocols were excluded from the analyses; one participant was excluded at VO_2_ and GE from the submaximal protocol with fixed inclination as this test could not be considered submaximal (the subject reached 95% of his VO_2peak_ during the protocol).

### Procedure

The participants prepared for the tests according to the instructions described earlier [17]. This meant that each participant arrived in the laboratory at the same time of day for all tests (G3 VO_2peak_, submaximal incremental test with fixed speed, submaximal incremental test with fixed inclination). Over the 24 hours preceding the first test, each participant was instructed to eat his normal diet for preparing for a sprint competition, and the subjects replicated this diet before the second and third tests. Subjects arrived for testing in a rested and hydrated state, at least 2 hours postprandial and had avoided strenuous exercise, caffeine and alcohol in the 24 hours preceding the test sessions. Supplementation during the tests was restricted to 500 mL of a sports drink (Powerade). The VO_2peak_ test was performed on a 4% inclined treadmill, and the speed was increased incrementally each minute by 2 km∙h^-1^ from 16 km∙h^-1^ to exhaustion. The mean of the three highest 10-s consecutive VO_2_ recordings at the end of the test was defined as VO_2peak_. VO_2peak_ was accepted when two of the following three criteria were reached: a respiratory exchange ratio (RER) above 1.10, a blood lactate concentration above 8 mmol∙L^-1^ and a plateau in VO_2_ with increasing exercise intensity [18].

Each participant performed a standardised warm-up, consisting of 10 min running at 60–70% of maximum HR on a motor-driven treadmill. After the warm-up, participants had a one-minute rest before the actual test started. The participants then rollerskied on a skiing treadmill for five minutes using the G3 technique. To exclude variations in rolling resistance, all subjects used the same pair of roller skis. The poles were provided with special carbide tips to prevent them from slipping on the treadmill belt. The participants performed, in randomised order, two submaximal protocols of G3 skating. In the first protocol, treadmill inclines of 7, 9 and 11% were used at a constant speed of 10 km∙h^-1^. In the second protocol, speeds of 14, 17 and 20 km∙h^-1^ were used and the incline was constant at 4%. Each step contained 2 x 5 minutes with SSP and LP. There was a 1-minute recovery between each 5-minute step in order to measure blood lactate concentration, register perceptual response and change poles. The subjects either started with the SSP and ended with the LP (SSP-LP, LP-SSP, SSP-LP), or started with the LP and ended with the SSP (LP-SSP, SSP-LP, LP-SSP). SSP pole length was 89 ± 0.6%, and LP pole length was 94 ± 0.5%, of body height.

During each test, VO_2_-uptake and HR were measured continuously. Furthermore, gross efficiency (GE) was calculated as external power divided by the total metabolic rate [19] and the formula for external power was calculated as the sum of power against gravity and friction:

Externalpower=m·g·v·[sin(α)+cos(α)·(μ)].

Here, *m* is the body mass, *g* the gravitational constant (9.81 m·s^-2^), *v* the treadmill speed, *α* the treadmill inclination and *μ* the frictional coefficient. The frictional coefficient was measured at 0.0237. Metabolic rate was calculated by using the $\dot{V}O_{2}$ and the associated respiratory exchange ratio (RER) from the last two minutes of each 5-minute interval together with the standard conversion tables [20].

Kinematic variables (cycle rate, cycle length) and knee angle were measured at the last step in each test (11% and 20 km∙h^-1^). The last steps were chosen for kinematic and angle analyses because these workloads are considered to be closest to competition conditions. Participants were always secured with a safety harness hanging from the ceiling, connected to the safety brake system of the treadmill.

### Instruments and measurements

The subjects skied on a treadmill (Rodby 3500ML, Södertalje, Sweden) using skating roller skis (SWENOR skate, standard resistance wheel 2, Trøsken, Norway) with Rottefella Performance skate bindings (Rottefella, Klokkarstua, Norway) and ski poles (Swix CT1, Lillehammer, Norway), and ran on a treadmill (Rodby 2500ML, Södertalje, Sweden).

Oxygen uptake was measured by an Oxycon Pro apparatus with a mixing chamber (Jaeger GmbH, Hochberg, Germany), using a 10-second interval for data storage. Before each test, the VO_2_ and VCO_2_ gas analysers were calibrated against both ambient air and a commercial mixture of high-precision gases (15.00 ± 0.04% VO_2_ and 5.85 ± 0.1% VCO_2_) (CareFusion gas GmbH, Hochberg, Germany) at the start of each test. The VO_2_ and VCO_2_ content of the ambient air was recorded and the flow meter was calibrated with a 3-L high-precision syringe (Hans Rudolph Inc., Kansas City, Missouri, USA). HR was measured with a heart rate monitor (Polar RC3GPS, Polar Electro OY, Kempele, Finland), using a five-second interval for data storage. The BIOSEN C-line Sport (EKF Diagnostic, Magdeburg, Germany) was used to measure blood lactate concentration from blood samples (20 μL) from the fingertip. The subjects’ rating of perceived exertion (RPE) was registered using the Borg (6–20) scale [21].

**Fig 1:**
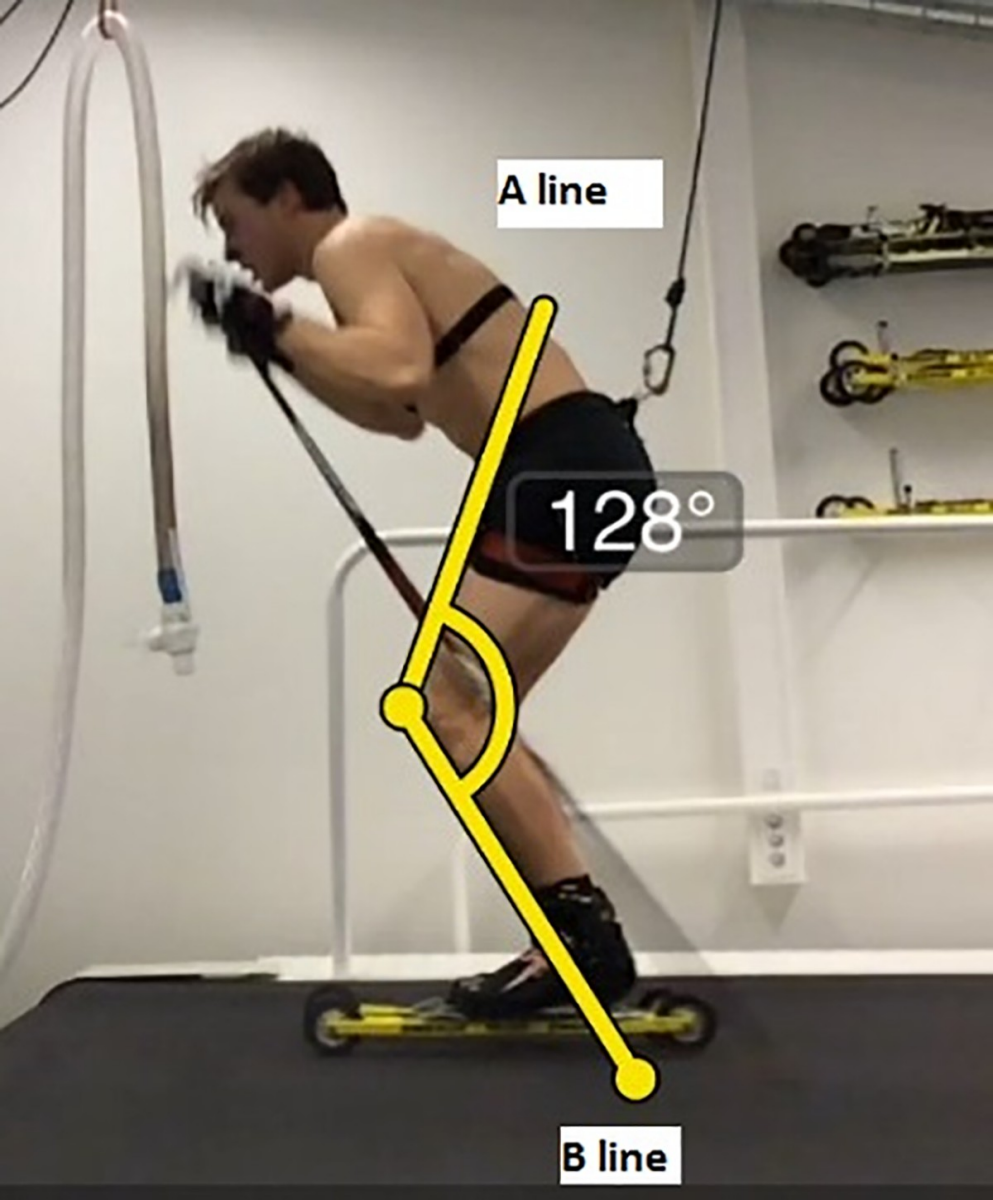
Illustration of knee angle measurements. Knee angle was determined at the lowest position where the legs were parallel just before left leg push. Lines A and B were drawn based on the front part of the thigh and shank. The skier shown in the figure signed a written consent form for usage of his image in this paper.

The skiers were 2-D video recorded during submaximal treadmill roller skiing by using an Apple iPad 4 (MD791KN/A USA) with 30 frames per second, and the video recordings were analysed for cycle length and cycle rate in the software Coach Eye (TechSmit Corp USA). The iPad was placed at 90° to the skiing direction on the skiers’ left side, 4.25 m from the centre of the skiing treadmill. Calculation of the average cycle characteristics was determined by timing 10 cycles and dividing them by 10, during the last 30 sec of the highest intensity in each condition (pole length, speed and inclination). Cycle time was taken from the time between two pole plants on the left side. Cycle length was calculated by multiplying the speed of the treadmill and the cycle time. Cycle rate was taken as the reciprocal of cycle time. Knee angle was taken at the lowest position, where the legs were parallel just before the left leg push. The two angle lines started at the position of the patella and touched the thigh and the leg (Fig 1).

The angles measured in the manner described above are not proper joint angles for the knee but were approximations which were judged to be more reproducible because estimations of hip, knee and ankle joint centres were not required. However, the reported angles were measured consistently for all skiers.

### Statistical analyses

The data were confirmed to be normally distributed with the Shapiro-Wilk test, and all results are presented as means ± standard deviation (SD), except for the perceptual responses; these are presented as median and interquartile range (IQR). To compare the effect of pole length on physiological and perceptual responses during submaximal treadmill roller skiing, two (LP versus SSP) x three (either inclines [7, 9, 11%] or velocities [14, 17, 20 km/h]) repeated measures ANOVA were performed. Post-hoc comparisons with Bonferroni correction were conducted to detect differences. A one-way repeated measures ANOVA was applied to compare the effect of pole length on cycle characteristics and knee angles at the highest inclination (11%) and at the highest speed (20 km∙h^-1^) in two separate analyses. Paired sample *t*-tests were applied when there were only two means to be compared (for example knee angle at 11% and 10 km∙h^-1^ with a comparison of SSP vs. LP). The effect size was reported as Cohen´s *d* (0 < d < 0.2 considered to be a very small, 0.2 < d < 0.5 a small, 0.5 < d < 0.8 a medium and d > 0.8 a large effect) [22]. The level of statistical significance was set at P ≤ 0.05. All statistical analyses were performed with the IBM SPSS Statistics for Windows, version 21.0 (IBM Corp., Armonk, NY, USA).

## Results

### Peak aerobic capacity and performance

The VO_2peak_ tested during uphill treadmill roller skiing at 4% inclination and a starting speed of 16 km∙h^-1^ averaged 68.7 ± 3.8 ml ∙ min^-1^ ∙ kg^-1^ and lasted for 418 ± 65 s. The average peak skiing speed was 26.6 ± 1.9 km∙h^-1^, and the corresponding values for RER, HR_peak_ and blood lactate concentration were 1.12 ± 0.07, 197.3 ± 5.3 beats·min^-1^, and 10.03 ± 2.28 mmol·L^-1^, respectively. The median (IQR) RPE score was 19 (1.3).

### Submaximal responses

Physiological, perceptual and kinematic variables for the two submaximal protocols are shown in Table 1 and Table 2. The VO_2_-uptake relative to treadmill roller skiing VO_2peak_ for the submaximal protocol with fixed speed at 10 km∙h^-1^ was 64 ± 4%, 74 ± 4% and 83 ± 4% at 7%, 9% and 11% inclination, respectively. The corresponding VO_2_-values for the submaximal protocol with fixed inclination at 4% were 64 ± 4%, 73 ± 4% and 85 ± 5% of VO_2max_ at 14 km∙h^-1^, 17 km∙h^-1^ and 20 km∙h^-1^, respectively. In both protocols, all physiological and perceptual variables increased with increasing intensity (i.e. increased inclination at fixed speed or increased speed at fixed inclination, all P < 0.001).

**Table 1:** Physiological and perceptual responses during uphill G3 roller skiing at three 5-minute submaximal workloads with increasing inclination at a fixed speed (10 km·h^-1^). Kinematic responses were obtained only during the steepest inclination (N = 10, mean ± SD).

	7%		9%		11%		ANOVA		
**Parameter**	SSP	LP	SSP	LP	SSP	LP	Pole length (PL)	Inclination (INC)	PL x INC
VO_2_ (ml·min^-1^·kg^-1^)	44.5 ± 1.5	44.0 ± 2.0	52.0 ± 2.1	51.0 ± 2.1	58.2 ± 2.0	56.6 ± 2.6*	F_1.9_ = 13.27##	F_2.18_ = 241.20###	F_2.18_ = 1.50
BLa (mmol·L^-1^)	1.76 ± 0.5	1.68 ± 0.5	2.58 ± 0.8	2.52 ± 0.8	4.35 ± 1.1	4.32 ± 1.2	F_1.9_ = 0.80	F_2.18_ = 92.05###	F_2.18_ = 0.044
RER	0.87 ± 0.3	0.88 ± 0.3	0.91 ± 0.4	0.91 ± 0.3	0.94 ± 0.3	0.94 ± 0.4	F_1.9_ = 0.94	F_2.18_ = 69.10###	F_2.18_ = 0.64
HR (beats·min^-1^)	156.7 ± 10.9	156.6 ± 11.1	173.4 ± 7.7	173.0 ± 8.2	184.6 ± 7.5	184.5 ± 7.0	F_1.9_ = 0.19	F_2.18_ = 158.74###	F_2.18_ = 0.27
^a^RPE (6–20)	9.5 (3.3)	10.5 (4.0)	13.0 (1.3)	13.0 (1.3)	16.0 (1.5)	16.0 (2.0)	F_1.9_ = 0.10	F_2.18_ = 99.62###	F_2.18_ = 1.04
Work rate (W)	186 ± 12		225 ± 14		265 ± 17				
Metabolic rate (W)	1103 ± 82	1079 ± 80	1292 ± 83	1274 ± 83	1458 ± 91	1418 ± 112**	F_1.9_ = 5.52#	F_2.18_ = 123.65###	F_2.18_ = 0.47
Gross efficiency (%)	17.0 ± 0.6	17.2 ± 0.8	17.5 ± 0.8	17.9 ± 0.8	18.2 ± 0.7	18.8 ± 1.0*	F_1.9_ = 14.08##	F_2.18_ = 20.91###	F_2.18_ = 0.60
Cycle length (m)					2.88 ± 0.1	2.89 ± 0.1			
Cycle rate (Hz)					0.96 ± 0.05	0.96 ± 0.05			
Knee angle (°)					126 ± 8	132 ± 7*			

SSP = self-selected pole length; LP = longer pole length (SSP + 7.5 cm); VO_2_ = oxygen uptake; BLa = blood lactate concentration; RER = respiratory exchange ratio; HR = heart rate; RPE = ratings of perceived exertion.

^a^Presented as median and inter quartile range (IQR).

* Significant difference between the two pole lengths at the same inclination: *P < 0.05

**P < 0.01.

# Main effect of pole length and main effect of inclination: #P < 0.05

## P < 0.01

### P < 0.001.

**Table 2:** Physiological and perceptual responses during uphill G3 roller skiing at three 5-minute submaximal workloads with increasing speed at a fixed inclination (4%). Kinematic responses were obtained only during the highest speed (N = 10, mean ± SD).

	14 km∙h^-1^		17 km∙h^-1^		20 km∙h^-1^		ANOVA		
Parameter	SSP	LP	SSP	LP	SSP	LP	Pole length (PL)	Speed (SP)	PL x SP
^1^VO_2_ (ml·min^-1^·kg^-1^)	44.8 ± 2.4	43.5 ± 3.1*	50.3 ± 1.6	50.1 ± 1.9	59.1 ± 2.5	57.9 ± 2.0**	F_1.8_ = 12.59##	F_2.16_ = 365.06###	F_2.16_ = 1.32
BLa (mmol·L^-1^)	1.72 ± 0.56	1.71 ± 0.58	2.31 ± 0.68	2.31 ± 0.60	4.16 ± 1.25	3.87 ± 0.94	F_1.9_ = 1.18	F_2.18_ = 103.07###	F_2.18_ = 2.91
RER	0.89 ± 0.03	0.89 ± 0.04	0.91 ± 0.03	0.91 ± 0.04	0.95 ± 0.04	0.94 ± 0.03	F_1.9_ = 2.03	F_2.18_ = 41.64###	F_2.18_ = 0.06
HR (beats · min^-1^)	151.7 ± 13.0	152.9 ±13.0	169.5 ± 11.4	169.6 ± 8.6	183.2 ± 7.7	181.9 ± 7.7	F_1.9_ = 0.000	F_2.18_ = 151.96###	F_2.18_ = 1.72
^a^RPE (6–20)	10.5 (3.5)	10.0 (2.3)	13.0 (2.0)	13.0 (2.0)	16.0 (1.5)	16.0 (1.0)	F_1.9_ = 0.14	F_2.18_ = 108.0###	F_2.18_ = 0.57
Work rate (W)	177 ± 11		215 ± 14		253 ± 16				
Metabolic rate (W)	1109 ±87	1070 ± 91	1276 ± 127	1244 ± 75	1504 ± 162	1454 ± 94	F_1.9_ = 3.63	F_2.18_ = 169.28###	F_2.18_ = 0.37
^1^Gross efficiency (%)	16.0 ± 0.9	16.6 ± 1.2*	16.9 ± 1.1	17.5 ± 0.7	16.9 ± 1.3	17.6 ± 0.8*	F_1.8_ = 5.95#	F_2.16_ = 6.77##	F_2.16_ = 0.15
Cycle length (m)					5.67 ± 0.32	5.68 ± 0.42			
Cycle rate (Hz)					0.98 ± 0.06	0.98 ± 0.08			
Knee angle (°)					129 ± 3	135 ± 6**			

SSP = self-selected pole length; LP = longer pole length (SSP + 7.5 cm); VO_2_ = oxygen uptake; BLa = blood lactate concentration; RER = respiratory exchange ratio; HR = heart rate; RPE = ratings of perceived exertion.

^1^N = 9.

^a^Presented as median and inter quartile range (IQR).

* Significant difference between the two pole lengths at the same inclination: *P < 0.05

**P < 0.01.

# Main effect of pole length and main effect of speed: #P < 0.05

## P < 0.01

### P < 0.001.

For the protocol with fixed speed at the steepest inclination (11%), the VO_2_ was lower at 56.6 vs. 58.2 ml·min^-1^·kg^-1^ (P = 0.005, Cohen´s *d* = 0.70) and the GE was higher (18.8% vs. 18.2%, P = 0.012, Cohen´s *d* = 0.71) with LP than with SSP. At the same inclination, the knee angle was 4.8% greater with LP than with SSP (P = 0.050, Cohen´s *d* = 0.80). At 9% inclination, there was a tendency towards lower VO_2_-uptake (P = 0.056, Cohen´s *d* = 0.48) and higher GE (P = 0.059, Cohen´s *d* = 0.50) with LP.

For the protocol with fixed inclination, the VO_2_-uptake was lower (43.5 vs. 44.8 ml·min^-1^·kg^-1^, P = 0.050, Cohen´s *d* = 0.47) and GE was higher (16.6% vs. 16.0%, P = 0.03, Cohen´s *d* = 0.57) with LP than with SSP at the lowest speed of 14 km∙h^-1^. At the highest speed of 20 km∙h^-1^, the VO_2_-uptake was lower (57.9 vs. 59.1 ml·min^-1^·kg^-1^, P = 0.01, Cohen´s *d* = 0.53), GE was higher (17.6% vs. 16.9%, P = 0.03, Cohen´s *d* = 0.64) and the knee angle was 5.5% greater (P = 0.003, Cohen´s *d* = 1.3) with LP, when compared to SSP.

## Discussion

The primary aim of this study was to investigate the effect of pole length on physiological and perceptual responses as a result of increasing speed and inclination for submaximal roller- skiing with the G3 ski skating sub-technique. The main findings in the current study were as follows: 1) LP induced lower VO_2_-uptake and higher GE in the two highest submaximal workloads, i.e. at 11% inclination and at 20 km∙h^-1^, compared to SSP. 2) At 4% inclination and at the lowest speed of 14 km∙h^-1^, the VO_2_-uptake was also lower and GE higher with LP compared to SSP. 3) The participants’ RPE on SSP and LP at all conditions were not significantly different. 4) The LP showed a greater knee angle at the two highest submaximal workloads compared to SSP conditions. 5) Additionally, there were no significant differences in cycle characteristics between SSP and LP at the two highest submaximal workloads.

### Effect of pole length on physiological and perceptual responses

Our findings in this particular study are in line with earlier investigations that claimed that longer poles in classic DP of up to ~ 90% of body height reduced the VO_2_-cost [11, 12, 14]. In the present study, the skiers were tested in the G3 ski skating sub-technique, and since it is assumed that performance in DP and G3 are limited by the same physiological and biomechanical factors with respect to at least upper-body work [9, 10], it is reasonable to assume that LP in G3 have the same advantages. At the two highest submaximal workloads (4% inclination and 20 km∙h^-1^, 11% inclination and 10 km∙h^-1^) the skiers in our study had a lower VO_2_-uptake and a higher GE when they used LP. The reason for this may be the biomechanical and muscular advantages of a more extended knee angle found in the lowest position. We only had rough estimations of knee angle in our study; however, with a greater knee angle in the skier´s lowest position, the skiers may end up in a more upright posture with less vertical displacement of COM. In addition, the effect of lower VO_2_-cost and higher GE due to LP than SSP was more pronounced in the steep uphill protocol than in the high speed protocol. Considered together with the interesting findings on DP of Losnegard [11] and Carlsen [12], it seems that the benefit of longer poles increases with steeper uphill terrain. This may be due to greater propulsive force, as longer poles allow the skier to use the upper body and body mass more effectively [15, 23]. Since most of the racing time is spent on uphill sections during a race and the greatest time differences between skiers occur on uphills [4], the novel findings in our study indicate that LP in G3 may enhance uphill performance and significantly influence race outcomes. However, in a more upright position using longer poles, the area of the skier might be larger and therefore also the air drag. Due to the low speed in uphill terrain, this would have a marginal or non-existing effect on our results but should be considered in flatter terrain where higher speeds are employed.

The knee-extension flexion pattern, performed from a higher position when LP were used, may be related to the lower VO_2_-cost. The SSP conditions showed that the skier was brought into positions where the external moment arm in the knee joint becomes greater than in LP conditions. The less extended knee joint at the lowest position before the kick starts with SSP will lead to more muscular loading, which could also lead to higher VO_2_-cost. Since the VO_2_-cost was lower and no differences in cycle characteristics were measured in this study, LP produces speed effectively even with the knee joint more extended than in the SSP condition, which may also be due to a more effective use of the upper body. The reason for the lower VO_2_-cost with longer poles in the research of DP cannot solely be explained by the reduced vertical displacement of COM. The reason for this is that the differences of COM between long and short poles are relatively small (1cm) [11, 12]. On the other hand, it is important not to underestimate small differences, in for example knee angle, in endurance sports like XC skiing, since every movement is repeated many times. Interestingly, skiers, even at the highest international level, have not utilised the potential of longer poles, approved by the FIS rules (FIS §343.8.2). However, the translation of our results to on-snow G3 skiing should be further investigated, and future studies are warranted to better understand which mechanisms may play a part in explaining the reduced physiological cost of uphill ski skating with longer poles.

The 4% and 14 km∙h^-1^ conditions also showed a lower VO_2_-cost and greater GE for LP. This metabolic rate corresponds to intensity zone 1 (I1) and is the most used training intensity zone for XC skiers. The 4% and 17 km∙h^-1^ conditions correspond to a metabolic rate at intensity zone two (I2) which is the training intensity zone XC skiers try to reduce to avoid fatigue in daily training [24]. The volume of specific training at the lowest (I1) and highest submaximal (I3) workloads (inclination and speed) may be the reason for a more effective use of LP in these two conditions (I1 and I3). However, there was no significant difference between SSP and LP at 4% and 17 km∙h^-1^.

During G3 skiing, the differences in cycle characteristics between SSP and LP were only measured at the two highest submaximal workloads (10 km∙h^-1^ and 11% inclination, and 4% inclination and 20 km∙h^-1^). No significant effect of pole length on cycle characteristics was found. In earlier studies [11, 12, 14], it was shown that pole length affected both kinematics and kinetics in DP. In these studies, increased pole length resulted in longer ground contact times, increased cycle length and reduced poling rate, which led to a more energetic and efficient poling technique. One reason for not finding differences in cycle characteristics between different pole lengths in the G3 technique in the current study may be that the leg push-off compensates in skate skiing, which is not possible when merely DP. Another explanation may be that we did not measure at maximal workloads, in contrast to the DP research.

The participants reported no significant differences in RPE with the use of LP and SSP in any of the three submaximal workloads. This corresponds well with our findings of no differences in RER and BLa between conditions. However, these findings contrast with anecdotes from the XC skiing community about the disadvantage of longer poles in a slower pole recovery phase and the aim to ski with ‘low shoulders’ in the repositioning phase, in addition to the fact that longer poles have increased mass and increased moment of inertia.

After testing, the skiers in this study did not give any negative feedback related to the use of LP compared to SSP. The performance in XC skiing will always be compromised by choice of sub-technique and equipment due to changing snow, weather and track conditions during a race. Hence, in optimal conditions and with practice, longer poles may be a suitable strategy in the G3 technique to enhance performance.

### Strengths, limitations and practical applications

Standard test methodology of physiology and RPE was utilised to evaluate the effects of pole length in the G3 technique in uphill skate skiing. Data in this study indicate that skiers might consider experimenting with longer poles in skate skiing to increase their performance. However, a direct translation to on-snow skiing and competitions (e.g. time trials) needs to be established in future research. In the lab, the measurement of kinematics, COM, range of motion and angles of the important joints in the skiing sub-techniques in particular should be analysed further with appropriate equipment and methods. Such information will enhance understanding of how VO_2_-cost is influenced by pole length. Furthermore, the ~2 ml · min^-1^ · kg^-1^ lower VO_2_-cost with LP on the uphill section has to be seen from the perspective that the reduced VO_2_-cost can be used to increase performance in cross-country skiing. However, some uphills in the world cup tracks are so steep that they probably are still best climbed, even for the strongest skiers, by using the G2 technique. However, considering that these steep uphills form a small part of the track, long poles could still provide a total better performance despite being a disadvantage on such terrain. The possible disadvantage of using longer poles on these steep uphill sections is unlikely to be so extreme as to exclude a possible effective use of the G2 technique. There is also a possibility that long poles may not be as stiff as shorter ones, which may lead to lower force transfer to the ground and forward propulsion. However, we do not know if long poles have any disadvantages in the G2 technique or in the issues mentioned above; this must therefore be further investigated under snow conditions. Long poles can also be a disadvantage in mass starts, sprints and relays because of the increased risk of broken poles due to a slightly wider pole plant. A shortcoming in this study is that we were unable to determine whether even longer poles would be still more beneficial or if the effect would be reduced. Further, we suggest a future long-term training study to investigate the effect of long poles. To evaluate training adaptations and the effect of long poles, an on-snow time trial performance test should be performed. The vast majority of research on this topic has been conducted in classic skiing; the present study therefore had to rely on this knowledge and use a similar methodological approach, since this is the first scientific work on this topic in skate skiing.

## Conclusions

The novel finding of this study is the superiority of longer poles over self-selected poles in G3 uphill ski skating sub-technique in terms of gross efficiency and VO_2_-cost both on uphill and at high speed on flatter terrain. Moreover, these results were associated with a more extended knee angle in the lowest position when using longer rather than self-selected poles. This latter finding may indicate that skiers have less vertical displacement when using longer poles, which can, at least partly, explain the lower VO_2_-cost and higher gross efficiency. While skier ratings of perceived exertion were not different between pole lengths at any of the submaximal workloads, clear differences of economy were observed. It is likely that cross-country skiers who choose longer poles rather than the typically preferred pole length have a modest metabolic advantage in G3 skating. Future studies should examine to what extent pole ground contact time and pole force effectiveness could explain the benefits of pole length in skating, and whether our findings would apply during outdoor on-snow skiing where air drag also plays a role.

## Supporting information

S1 File Supporting data. (XLSX)
